# Whole Exome Sequencing Analysis in Fetal Skeletal Dysplasia Detected by Ultrasonography: An Analysis of 38 Cases

**DOI:** 10.3389/fgene.2021.728544

**Published:** 2021-09-10

**Authors:** Ying Peng, Shuting Yang, Xiaoliang Huang, Jialun Pang, Jing Liu, Jiancheng Hu, Xinzhao Shen, Chengyuan Tang, Hua Wang

**Affiliations:** ^1^Prenatal Diagnosis Center, National Health Commission Key Laboratory of Birth Defects Research, Hunan Provincial Maternal and Child Health Care Hospital, Changsha, China; ^2^Berry Genomics Corporation, Beijing, China; ^3^Department of Nephrology, Hunan Key Laboratory of Kidney Disease and Blood Purification, The Second Xiangya Hospital, Central South University, Changsha, China

**Keywords:** skeletal dysplasia, prenatal diagnosis, whole-exome sequencing, ultrasound examination, fetus

## Abstract

**Background:** Skeletal dysplasias (SDs) are a heterogeneous group of genetic disorders that primarily affect bone and cartilage. This study aims to identify the genetic causes for fetal SDs, and evaluates the diagnostic yield of prenatal whole-exome sequencing (WES) for this disorder.

**Methods:** WES was performed on 38 fetuses with sonographically identified SDs and normal results of karyotype and single nucleotide polymorphism (SNP) analysis. Candidate variants were selected by bioinformatics analysis, and verified by Sanger sequencing.

**Results:** WES revealed pathogenic or likely pathogenic variants associated with SDs in 65.79% (25/38) of fetuses, variants of uncertain significance (VUS) in SDs-related genes in 10.53% (4/38) cases, and incidental findings in 31.58% (12/38) fetuses. The SDs-associated variants identified in the present study affected 10 genes, and 35.71% (10/28) of the variants were novel.

**Conclusion:** WES has a high diagnostic rate for prenatal SDs, which improves pregnancy management, prenatal counseling and recurrence risk assessment for future pregnancies. The newly identified variants expanded mutation spectrum of this disorder.

## Introduction

Skeletal dysplasias (SDs) are a heterogeneous group of disorders that affect bone and cartilage and are characterized by abnormal shape, growth, and integrity of the skeleton. Its prevalence is estimated to be about 1 of 4,000–5,000 live births (Orioli et al., 1986; Stoll et al., 1989), and account for nearly 10% of fetal structural malformations detected by ultrasonography (Tang et al., 2020). Currently, SDs comprise 461 types of diseases that are categorized into 42 groups, and pathogenic variants in 437 different genes have been linked to 92% (425/461) of these diseases (Mortier et al., 2019). In addition, pathogenic variants in an individual gene can cause different phenotypes, and similar phenotypes can be resulted from variants in different genes. The high genetic and phenotypic diversity make prenatal diagnosis of SDs by ultrasound or magnetic resonance imaging challenging (Schramm et al., 2009; Geister and Camper, 2015), while molecular diagnosis offers the possibility of identifying the specific entity underlying SDs.

The conventional molecular diagnosis strategies include karyotyping and chromosomal microarray analysis (CMA). Karyotyping has a diagnostic yield of around 30% in fetuses with sonographically identified structural birth defects (Zhang et al., 2017), and CMA provides an additional diagnostic yield of 4–6% in fetuses with an ultrasound anomaly and normal karyotype (Wapner et al., 2012). Thus, using these molecular diagnosis methods, less than half of fetuses with structural anomalies can be diagnosed. In addition, SDs are mainly monogenic disorders (Geister and Camper, 2015; Mortier et al., 2019), and therefore molecular diagnosis methods with high resolution is need for their diagnosis. Next-generation sequencing (NGS) provides the deep, high-throughput, and in-parallel DNA sequencing. Whole-exome sequencing (WES) is a NGS method that sequences exons that contain > 85% of the genetic variants associated with human disease phenotypes (Rabbani et al., 2014). Two recent large-scale prospective studies in fetus with structural anomalies revealed that WES provided an additional diagnostic yield of 8–10% in fetuses with an ultrasound anomaly and normal karyotype and CMA results, and the detection rate is strongly correlated with the number of fetal anomalies (Lord et al., 2019; Petrovski et al., 2019). Lord et al. demonstrated that WES had a diagnosed yield of 15⋅4%(10/65) in fetuses with skeletal anomalies and normal karyotype and CMA results (Lord et al., 2019). In a cohort of 28 chromosomally normal fetuses with SD, Liu et al. (2019) revealed that 75% (21/28) of cases were detected with mutations in genes related to skeletal diseases by WES.

In the present study, WES was performed to identify genetic causes for 38 fetuses with sonographically identified SDs and normal results of karyotype and single nucleotide polymorphism (SNP) array analysis, and to evaluate the diagnostic rate of WES for prenatal SDs.

## Materials and Methods

### Subjects and Sample Collection

The study was approved by the ethics committee of Hunan Provincial Maternal and Child Health Care Hospital. All parents agreed to participate in the study and provided signed informed consent.

**FIGURE 1:**
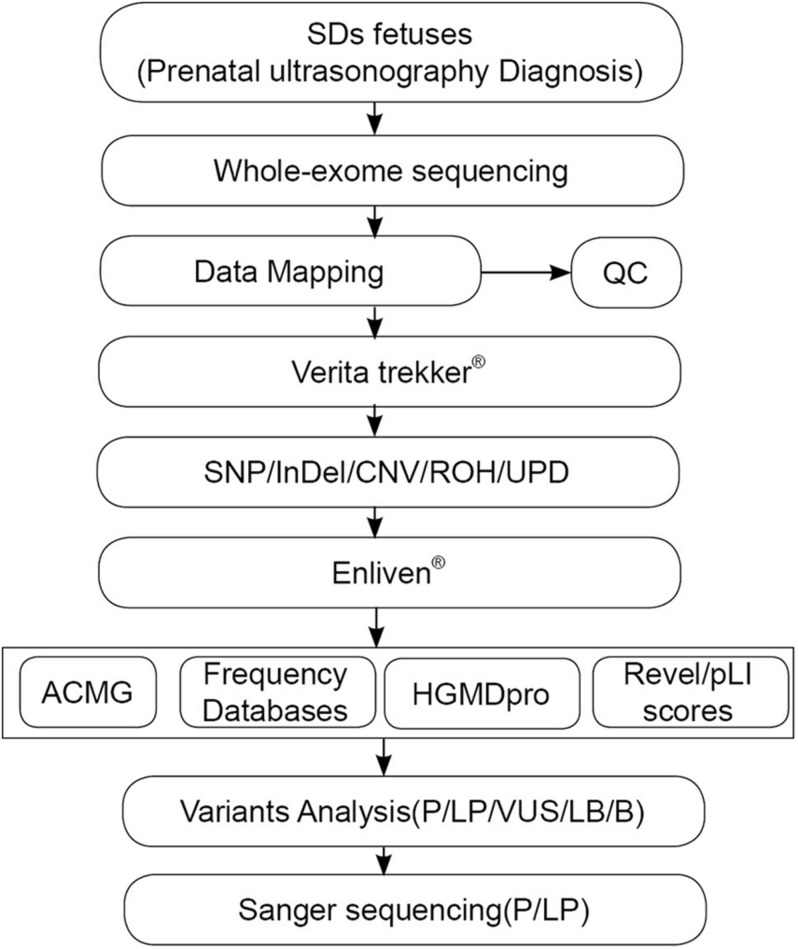
The overall experimental workflow in the present study. ACMG, American College of Medical Genetics and Genomics; Verita trekker^®^ and Enliven^®^ represent patented software of Berry Genomics; CNV, copy number variation; HGMD, Human Gene Mutation Database; InDel, insertion/deletion; Mt, mitochondrial variation; QC, quality control; ROH, runs of homozygosity; SNP, single nucleotide polymorphism; WES, whole–exome sequencing.

Thirty-eight fetuses diagnosed as SDs by prenatal ultrasonography at our center from 2016 to 2019 were enrolled in this study. The sonographic criteria incudes: (1) the presence of short limb deformities in which fetal femur length (FL) < −2 SD or FL below the 5th centile of our reference ranges at midtrimester ultrasonography), and/or (2) the presence of other skeletal anomalies including various deformities, finger/toe deformities, missing fingers/toes, and/or absence of upper/lower limbs. The overall study workflow was shown in Figure 1. The clinical data and the results of prenatal ultrasound examination are shown in Table 1. The fetal amniotic fluid, umbilical cord blood or skin tissue of aborted fetuses, and peripheral blood of family members were collected (Table 1).

**TABLE 1:** Prenatal (ultrasound) phenotype and genotype information for the cohort (*n* = 38).

Case	GA weeks	Ultrasound findings						Sample	Sequencing results by clinical WES			Clinical diagnosis	Pregnancy outcome
		Fetal biometric measurements (Z-scores)				AFI (cm)	Other skeletal/non-skeletal anomalies		Variant/heterozygosity/inheritance	ACMG Level	HGMD		
		BPD	HC	AC	FL								
1	17	−0.14	0.62	−1.03	−5.53	10.8	Short limbs, narrow thorax, collapse of sternum	Amniotic fluid	*FGFR3* c.742C > T p.Arg248Cys	P (PS1 + PS2 + PM1 + PM2 + PP3)	DM	Thanatophoric dysplasia, type I	Termination
									Het/AD/*de novo*				
2	17^+1^	2.25	2.25	0.47	−3.59	8.5	Short limbs, narrow thorax, collapse of sternum, curved femurs	Amniotic fluid	*FGFR3* c.742C > T p.Arg248Cys	P (PS1 + PM1 + PM2 + PM6 + PP3)	DM	Thanatophoric dysplasia, type I	Termination
									Het/AD/*de novo*				
3	23^+^	2.62	3.56	−0.31	−8.48	13.5	$short limbs, narrow thorax	Skin tissue	*FGFR3* c.746C > G	P (PS1 + PM1 + PM2 + PM6 + PP3)	DM	Thanatophoric dysplasia, type I	Termination
									p.Ser249Cys				
									Het/AD/*de novo*				
4	26^+^	0.56	0.16	−1.29	−12.14	24.3	Short limbs, skull deformation and out of flatness, collapse of sternum	Skin tissue	*FGFR3* c.746C > G	P (PS1 + PM2 + PM6 + PP3 + PP4)	DM	Thanatophoric dysplasia, type I	Termination
									p.Ser249Cys				
									Het/AD/*de novo*				
5	33^+1^	2.29	2.38	1.48	−4.52	21.4	Short limbs, skull deformation and out of flatness, narrow thorax, collapse of sternum	Umbilical cord blood	*FGFR3* c.1144G > A p.Gly382Arg	P (PS1 + PM2 + PM6 + PP3 + PP4)	NA	Achondroplasia	Termination
									Het/AD/*de novo*				
6	32^+^	1.45	1.68	−0.18	−6.05	27.9	Short limbs, skull deformation and out of flatness, collapse of sternum, enlarged lateral ventricle, curved femurs	Umbilical cord blood	*FGFR3* c.1144G > A p.Gly382Arg Het/AD/*de novo*	P (PS1 + PM2 + PM6 + PP3 + PP4)	NA	Achondroplasia	Termination
7	32^+^	3.53	3.66	1.47	−4.63	36.9	Short limbs, skull deformation and out of flatness, collapse of sternum, flattened nasal bridge	Umbilical cord blood	*FGFR3* c.1144G > A p.Gly382Arg Het/AD/*de novo*	P (PS1 + PM2 + PM6 + PP3 + PP4)	NA	Achondroplasia	Termination
8	19^+^	4.67	4.67	1.89	−5.29	11	Short limbs, narrow thorax, collapse of sternum, horseshoe kidney	Amniotic fluid	*FGFR3* c.1118A > G	P (PS1 + PM1 + PM2 + PM6 + PP3)	DM	Thanatophoric dysplasia, type I	Termination
									p.Tyr373Cys				
									Het/AD/*de novo*				
9	37^+2^	0.8	0.01	2.77	−7.93	16.1	Short limbs, skull deformation and out of flatness	Skin tissue	*FGFR3* c.1138G > A	P (PS1 + PM2 + PM6 + PP3 + PP4)	DM	Achondroplasia	Termination
									p.Gly380Arg				
									Het/AD/*de novo*				
10	31^+1^	1.22	0.66	−0.85	−2.99	21.3	Short limbs, skull deformation and out of flatness	Umbilical cord blood	*FGFR3* c.1138G > A	P (PS1 + PM1 + PM2 + PM6 + PP3)	DM	Achondroplasia	Termination
									p.Gly380Arg				
									Het/AD/*de novo*				
11	33^+5^	1.75	1.18	−0.1	−4.17	20.4	Short limbs, skull deformation and out of flatness, collapse of sternum, uneven arrangement of fetal spine	Umbilical cord blood	*FGFR3* c.1138G > A	P (PS1 + PM2 + PM6 + PP3 + PP4)	DM	Achondroplasia	Termination
									p.Gly380Arg				
									Het/AD/*de novo*				
12	31^+2^	1.06	0.93	0.01	−3.24	17.6	Short limbs, thorax narrow	Umbilical cord blood	*FGFR3* c.1138G > A	P (PS2 + PS1 + PS3 + PS4 + PP1 + PM2 + PP4)	DM	Achondroplasia	Termination
									p.Gly380Arg				
									Het/AD/*de novo*				
13	40^+2^	1.48	1.43	1.13	−3.31	8.9	Short limbs, curved femur	Umbilical cord blood	*FGFR3* c.1620C > G	P (PS2 + PS1 + PS3 + PS4 + PP1 + PM2 + PP4)	DM	Hypochondroplasia	
									p.Asn540Lys				
									Het/AD/*Mat*				
								Skin tissue	*COMP* c.1255-5C > T	LP (PVS1 + PM2)	NA	Epiphyseal, dysplasia, multiple, 1	
									Het/AD/Mat				
14	20^+4^	−0.48	−0.77	−0.84	−6.39	15.7	Short limbs, curved femur	Skin tissue	*COL1A1*	LP (PM2 + PM6 + PP3 + PP4)	DM	Osteogenesis imperfecta types I or III	Termination
									c.1192G > T				
									p.Gly398Cys				
									Het/AD/*de novo*				
15	24^+1^	−3.37	−2.12	−5.67	−13.16	11.7	Short limbs, skull deformation and out of flatness, thorax narrow, collapse of sternum	Skin tissue	*COL1A1*	LP(PM2 + PS2)	NA	Osteogenesis imperfecta types I or III	Termination
									c.1373G > A				
									p.Gly458Glu				
									Het/AD/*de novo*				
16	21^+5^	−2.92	−3.01	−4.42	−9.85	7	Short limbs, skull deformation and out of flatness, ventricular septal defect, pericardial effusion	Skin tissue	*COL1A2*	LP (PM2 + PS2)	NA	Osteogenesis imperfecta types I or III	Termination
									c.2422G > T				
									p.Gly808Cys				
									Het/AD/*de novo*				
17	26^+4^	1.85	2.57	−0.02	−7.12	24.3	Short limbs, bilateral foot inversion,	Skin tissue	*COL2A1*	LP (PS1 + PM2 + PM6 + PP3)	NA	Achondrogenesis, typeII	Termination
									c.1358G > T				
									p.Gly453Val				
									Het/AD/*de novo*				
18	23^+3^	−0.47	−0.08	−0.22	−6.28	17.4	Short limbs, curved femur	Umbilical cord blood	*COL2A1*	LP (PM2 + PM6 + PP3 + PP4)	NA	Achondrogenesis, typeII	Termination
									c.3571G > A				
									p.Gly1191Arg				
									Het/AD/*de novo*				
19^*^	32^+^	−0.85	−1.84	−1.15	−0.85	13.2	Lumbar spinal stenosis, lateral ventricle enlargement	Skin tissue	*COL2A1*	P (PS1 + PM2 + PM6 + PP3 + PP4)	NA	Achondrogenesis, type II	
									c.1419 + 5G > T				
									Het/AD/*de novo*				
20	24^+^	−2.08	−2.08	−2.34	−3.58	14	Short limbs, curved left femurs	Amniotic fluid	*COL2A1*	VUS (PS2 + BP7)	NA	Achondrogenesis, type II	Termination
									c.17C > A				
									p.Ala6Asp				
									Het/AD/*de novo*				
21^*^	16^+6^	3.6	−	−	0.8	2	Narrow thorax, collapse of sternum, abduction of lower limb	Skin tissue	*TRIP11*	P(PVS1 + PM2 + PP3 + PP4)	NA	Achondrogenesis, type 1A	Termination
									c.5056 + 1G > C				
									Hom/AR/Pat/Mat				
22^*^	13^+6^	2.08	−	−	0.68	3.7	Thickening of fetal body subcutaneous tissue, neck hydrocystoma, curved long bones, bilateral foot inversion	Skin tissue	*SOX9*	P(PVS1 + PM2 + PP3 + PP4)	NA	Acampomelic campomelic dysplasia	Termination
									c.916dup				
									p.Val306Glyfs^*^272				
									Het/AD/*de novo*				
23	23^+^	0.95	0.67	0.85	−4.64	12.7	Short limbs, skull deformation and out of flatness, narrow thorax	Skin tissue	*LBR* c.1757G > A	P(PM3 + PM1 + PM2 + PP3 + PS3)	NA	Greenberg skeletal dysplasia	Termination
									(p.Arg586His)				
									Het/AR/Mat				
									*LBR* c.194delG	P (PM3 + PM1 + PM2 + PP3 + PS3)	NA		
									(p.Gly65Valfs^*^53)				
									Het/AR/*de novo*				
24	38^+5^	−1.3	−0.13	0	−3.13	22.3	Short limbs, enhancement of gallbladder echo	Skin tissue	*IFT172*	LP (PM5 + PP4 + PS3 + PM3 + PM6)	NA	Short-rib thoracic dysplasia 10 with or without polydactyly	Termination
									c.1513C > T				
									(p.Arg505Trp)				
									Het/AR/Pat				
									*IFT172*	P (PM3 + PM1 + PM + PP3 + PS3)	NA		
									c.4540-5T > A				
									Het/AR/Mat				
25^*^	24^+^	2.04	1.63	0.63	0.75	27.7	Scaphocephaly, absence of bilateral thumbs, hypospadias, pericardial effusion, umbilical cord cyst	Skin tissue	*FIG4*	P (PM3 + PM1 + PM2 + PP3 + PS3)	NA	Yunis-varon syndrome	Termination
									c.573del				
									(p.Glu192Lysfs^*^2)				
									Het/AR/Pat				
									*FIG4*	P (PM3 + PM1 + PM2 + PP3 + PS3)	NA		
									c.2174dup				
									(p.Leu726Ilefs^*^7)				
									Het/AR/Mat				
26	25 +	−0.26	−1.63	−1.18	−10.18	13.7	Short limbs, narrow thorax, pleural and peritoneal effusion, bilateral renal pelvis and collection system expansion	Skin tissue	*DYNC2H1*	P (PM3 + PM1 + PM2 + PP3 + PS3)	NA	Short-rib thoracic dysplasia 3 with or without polydactyly	Termination
									c.3133C > T (p.Gln1045^*^)				
									Het/AR/Mat				
									*DYNC2H1*	VUS (PS1 + PM2 + PM6 + PP3 + PP4)	NA		
									c.6809G > A (p.Arg2270Gln)				
									Het/AR/Pat				
27^*^	30^+2^	0.27	0.02	−0.33	−0.81	11.9	Syndactyly on right hand, split hand malformation	Umbilical cord blood	*HOXD13*	VUS (PS1 + PM2 + PM6 + PP3 + PP4)	NA	Synpolydactyly 1	Termination
									c.328C > T				
									(p.Pro110Ser)				
									Het/AD/Pat				
28	19	−0.38	−0.47	−1.18	−3.81	15	short limbs	amniotic fluid	*IFITM5*	VUS (PM2 + PP3)	DM	Osteogenesis imperfecta	Termination
									c.119C > G				
									p.Ser40Trp				
									Het/AD/Pat				
29	36^+^	0.74	0.68	0.87	−5.28	28.6	Short limbs, flattened nasal bridge,	Skin tissue	–	–		–	Termination
30	25^+6^	−1.0	−1.25	−0.74	−2.57	16.2	Short limbs, curved femur,	Amniotic fluid	–	–		–	Termination
31	22^+2^	−0.18	−0.91	−3.09	−5.55	12.6	Short limbs, hyperechogenic of bilateral kidneys, intermittent nasal bone echoes, collapsed thorax, curved limbs	Skin tissue	–	–		–	Termination
32	17^+6^	0.21	0.44	0.99	−4.92	8.7	Short limbs, narrow thorax, collapse of sternum, pulmonary hypoplasia, curved long bones	Skin tissue	–	–		–	Termination
33^*^	31^+1^	−2.16	−2.68	−1.94	−1.06	11.6	Absence of fingers, hand cleft, partial absence of phalanges	Skin tissue	–	–		–	Termination
34^*^	31^+6^	0.93	0.23	−1.71	1.67	14	Absence of right humerus, curved long bones, spondylometaphyseal dysplasia	Skin tissue	–	–		–	Termination
35^*^	23^+0^	0.61	0.67	−1.63	0.30	17.5	Abnormalities of wrist joint, syndactyly on right hand, inversion of bilateral foot	Amniotic fluid	–	–		–	Termination
36	34^+1^	−0.09	0.07	−0.15	−2.07	11.5	Short limbs	Umbilical cord blood	–	–		–	Termination
37	19^+5^	−1.77	−0.95	−1.67	−9.14	7.4	Short limbs, curved femurs, inversion of bilateral foot	Skin tissue	–	–		–	Termination
38	32^+3^	0.9	1.5	0.12	−4.27	18.2	Short limbs	Umbilical cord blood	–	–	–		Termination

*The formula: Z-score = (XGA -MGA)/SDGA, where XGA is data from other populations at a known gestational age, MGA is the mean value for our population calculated from the reference equations at this gestational age, and SDGA is the SD associated with the mean value at the same gestational age from our population. The fetal biometric measurements of other populations at each gestation were expressed as Z-scores calculated with our reference equations.*

*GA: gestational age; BPD: biparietal diameter; HC: Head circumference; AC: Abdominal circumference; FL: Femur length; AFI: Amniotic fluid index; PVS: pathogenic very strong); PS: pathogenic strong, PM: pathogenic moderate; PP: pathogenic supporting); BA: benign stand-alone; BS: benign strong; BP: benign supporting.*

*HGMD: Human Gene Mutation Database (Professional Version 2020s4); DM: disease-causing mutation.*

*Het: heterozygosity; Hom: homozygosity; P:pathogenic; LP: likely pathogenic;VUS: unknown clinical significance; NA: not available; AD:autosomal dominant; AR: autosomal recessive.*

^*^Fetuses do not show short limbs.

### Whole-Exome Sequencing

#### Library Construction and Sequencing

Genomic DNA of the 38 fetuses and 7 pairs of parents were extracted using a Qiagen DNA Blood Midi/Mini kit (Qiagen GmbH, Hilden, Germany) as previously described (Peng et al., 2020). DNA was sheared into fragments of around 150 bp and blunt-ended followed by addition with deoxyadenosine at the 3′ ends. The DNA library was generated by ligating adaptors to the ends of double DNA strands that enable sequencing. The library was amplified by PCR and then hybridized to a pool of biotinylated oligo probes specific for exons. Streptavidin magnetic beads were used to capture DNA-probes hybrids. The hybrid products were eluted and collected, and were subsequently amplificated by PCR. The libraries were tested for enrichment by qPCR, and size distribution and concentration of the libraries were determined using an Agilent Bioanalyzer 2100 (Agilent Technologies, Santa Clara, CA, United States). For sequencing, the genomic DNAs were sequenced using NovaSeq 6000 platform (Illumina, San Diego, CA, United States) with 150 bp pair-end reads. Raw image files were processed using CASAVA (v1.82) for base calling and generating raw data.

#### Data Analysis

Adaptor sequences of the raw data were trimmed at the tails of reads using Cutadapt (v1.15) (Martin M. Cut adapt removes adapter sequences from high-throughput sequencing reads. EMBnet 17:10-12[J]), and then aligned to the human reference genome (hg19/GRCh37) with using Burrows–Wheeler Aligner tool. PCR duplicates were removed using Picard v2.4.1.^1^ Duplicated reads were marked by Picard (v2.4.1; see text footnote 1). Qualimap (v2.2.1) was used to calculate base quality metrics, genome mapping rate, and the coverage of targeted regions (Garcia-Alcalde et al., 2012). Indel realignment and variants [single nucleotide variants (SNVs) and indels] calling were performed using Verita Trekker^®^ Variants Detection System by Berry Genomics and the third-party software Genome Analysis Toolkit (GATK, v3.8)^2^ (Van der Auwera et al., 2013). Variant annotation and interpretation were conducted by ANNOVAR (Wang et al., 2010) and the Enliven^®^ Variants Annotation Interpretation System authorized by Berry Genomics. Annotation databases mainly included gnomAD^3^, the 1000 Genome Project^4^, Berry big data population database, dbSNP^5^, OMIM^6^, ClinVar^7^, HGMD^8^, and HPO^9^. The mean depth of coverage for whole exomes by WES was 100× and a mean of 97.5% of bases was covered by at least 20 reads.

#### Data Filtering and Diagnostic Interpretation of the Filtered Variants

Variants that have any of the following conditions were excluded, including: (1) sequencing depth is ≤ 10; (2) maximum population frequency was larger than 0.01 when referring to dbSNP build 142, the 1,000 Genomes Project and the ExAC Browser; (3) not in coding or exon–intron junction region; and (4) predicted as benign by SIFT, FATHMM, MutationAssessor, CADD, and PolyPhen-2. The remaining variants were identified as potential SNVs. The variants identified were then classified into to five categories according to the American College of Medical Genetics and Genomics (ACMG) recommendations of 2015 (Richards et al., 2015), including pathogenic, likely pathogenic, benign, likely benign, and variants of uncertain significances (VUSs). The ACMG guidelines define 28 criteria that are grouped into seven categories of evidence including pathogenic very strong (PVS), pathogenic strong (PS), pathogenic moderate (PM), pathogenic supporting (PP), benign stand-alone (BA), benign strong (BS), benign supporting (BA). Each of the seven categories includes one or multiple criteria (for example, PS has four criteria PS1–PS4), and each criterion is defined based on a variety of information including: (1) the type of variant and population frequency, (2) the patient’s overall clinical presentation and its concordance with the phenotype described in the literature, (3) the family history, the predicted mode of transmission, and the familial segregation, (4) the prevalence of the disease compared with the frequency of the variant in the general population, (5) the predicted effect of the variant by *in silico* predictive algorithms, and (6) the functional data available in the literature. By combining these criteria, a variant is classified into one of the five categories.

### Verification of Gene Mutations by Sanger Sequencing

The pathogenic or likely pathogenic mutations detected by WES were confirmed by Sanger sequencing.

## Results

### Clinical Findings

In this study, 75% (30/38) fetuses had short limb deformities, and the other fetuses (fetus 19, 21, 22, 25, 27, and 33–35) showed no short limb deformities. 29 pathogenic/likely pathogenic variants in SDs associated genes were found in 25 cases, including (Table 1). The ultrasound results of 13 cases with novel mutations were shown in Supplementary Figure 1.

### Genetic Variations

**FIGURE 2:**
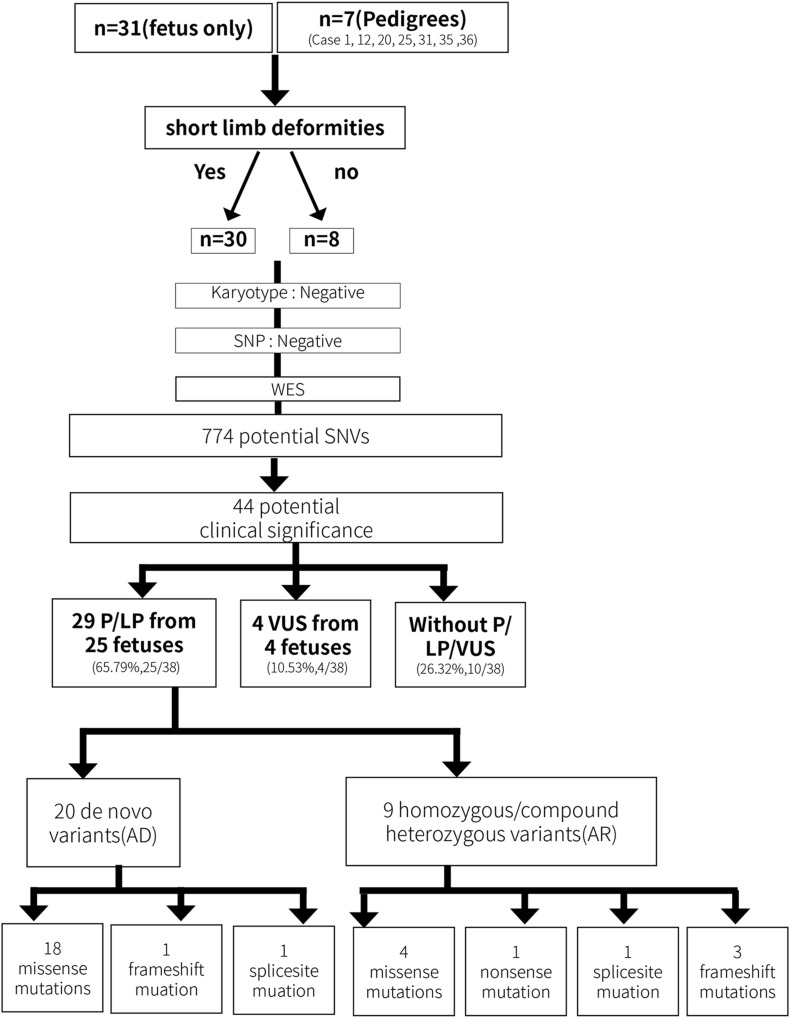
The overall experimental workflow for identifying genetic variations in the present study.

The karyotyping results were normal for the 31 fetuses (data not shown). WES was successfully performed on 31 fetus samples and 7 father–mother–proband samples (fetus 1, 12, 20, 25, 31, 35, 36 and their parents). The results were shown in Figure 2.

A total of 774 potential SNVs were initially identified, including 626 from the fetus only group and 148 from the fetus–mother–father trio group. Among them, 34 variants from the fetal proband-only group and 10 variants from the fetus–mother–father trio group were evaluated to be of potential clinical significance. Among the 44 variants, 22 pathogenic variants and 7 likely pathogenic variants from 25 fetuses were evaluated to be directly associated with SDs (Table 1 and Figure 2), thereby yielding a positive detection rate of 65.79% (25/38). 4 VUS in SDs-related genes were detected in 4 fetuses (Table 1 and Figure 2), yielding an VUS detection rate of 10.53% (4/38). Among the 29 SDs-related pathological/likely pathogenic variants, 20 were *de novo* variants that followed an autosomal dominant inheritance pattern, including 18 missense mutations, 1 frame-shift mutation, and 1 splice-site mutation, and 9 variants were functional homozygous or compound heterozygous mutations in accordance with their recessive patterns of inheritance, including 4 missense mutations, 1 nonsense mutation, 3 were frameshift mutations, and 1 splice site mutation (Table 1).

When categorizing the fetal samples into short-limb subgroup and non-short limb group, a definitive diagnosis was made in 70.0% (21/30) cases with short-limb and in 62.5% (5/8) cases without short-limb.

## Discussion

Skeletal dysplasias are a heterogeneous group of genetic disorders that account for nearly 10% of the fetal structural malformations (Tang et al., 2020). In this study, WES was performed on 38 fetuses with sonographically identified SDs and normal results of karyotype and SNP array analysis. Pathogenic/likely pathogenic variants were identified in 65.79% (25/38) of the fetuses, which indicates a high diagnostic yield of WES for prenatal molecular diagnoses of SDs. 10 novel variants identified in this study expand mutation spectrum of SDs and contribute to the genetic diagnosis and counseling of this disorder. This study also identified pathogenic variants during the WES data analysis that are beyond the scope of the SD for which the fetuses were prescribed WES tests, yielding an incidental detection rate of 31.58% (12/38). Current ACMG guidelines recommend the reporting of incidental findings in clinical exome and genome sequencing, specifically pathogenic variants in 59 medically actionable genes (Kalia et al., 2017), and therefore this high rate of incidental findings will present greater ethical challenges during genetic counseling.

Pathogenic variants from 437 different genes have been associated with SDs (Mortier et al., 2019). In the present study, 28 variants affecting 10 genes including *FGFR3, COL1A1, COL1A2, COL2A1, TRIP11, SOX9, LBR, IFT172, FIG4*, and *DYNC2H15* were found to be associated with the SDs (Table 1). Among the 25 fetuses of SDs with genetic diagnosis, 52% (13/25) of fetuses contained variants in *FGFR3*, 16% (4/25) of fetuses contained variants in *COL2A1*, and 8% (2/25) fetuses contained variants in *COL1A1.*The other 4 fetuses carried variants in one of the 6 genes including *TRIP11, SOX9, LBR, IFT172, FIG4*, and *DYNC2H1.* These findings are in line with the high genetic variability of SDs, and also highlights that pathogenic variants in *FGFR3* and collagen genes are the most common generic lesion for SDs.

**FIGURE 3:**
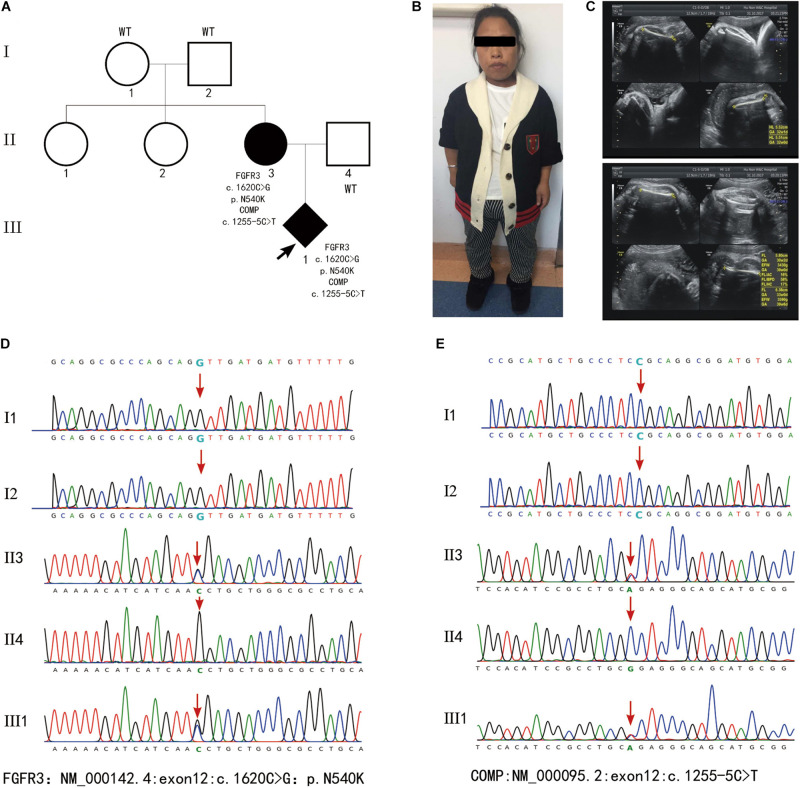
**(A)** Family pedigrees of fetus 13. **(B)** Picture of the mother who displayed short stature (135cm in height), slender limbs and lumbar anterior straighten deformity. **(C)** Images of ultrasound examination of fetus 13 at 40 weeks’ gestation revealed short limbs, curved femur. **(D,E)** Results of WES. Fetus 13 and the mother who was affected with SDs contained heterozygous mutation *FGFR3*:exon12:c.1620C > G:p.N540K and *COMP*:exon12:c.1255-5C > T.

Fibroblast growth factor receptor 3 (FGFR3) is one of four distinct membrane-spanning tyrosine kinases that serve as high-affinity receptors for a number of fibroblast growth factors and plays essential roles in skeletal development (Ornitz and Marie, 2015). Currently, variants in *FGFR3* have been associated with at least 10 different skeletal disorders (Mortier et al., 2019), and the distribution of the known disease-associated *FGFR3* germline mutations suggests there is no preferential type or location of mutations for a distinct disorder. In the present study, fetus 1 and 2 carried an identical variant c.742C > T (p.Arg248Cys) in the extracellular region, fetus 3 and 4 carried a variant c.746C > G (p.Ser249Cys) in the extracellular region, and fetus 8 carried mutation c.1118A > G (p.Tyr373Cys) in the transmembrane domain. These three mutation have been associated with thanatophoric dysplasia, type I (MIM_187600) (Brodie et al., 1999).The variant c.1144G > A (p.Gly382Arg) in fetus 5-7 and the variant c.1138G > A (p.Gly380Arg) in fetus 9–12 localize in the transmembrane domain, all of which are associated with achondroplasia (MIM_100800) (Hafner et al., 2006; Natacci et al., 2008). Fetus 13 inherited a heterozygous mutation c.1620C > G (p.Asn540Lys) in the TK-1 domain of FGFR3, which is a common cause of hypochondroplasia (OMIM_146000) (Mortier et al., 2000). Notably, a novel heterozygous *COMP* mutation c.1255-5C > T was also detected in fetus 13 and the mother, but not in the healthy family members (Figure 3). Variants in *COMP* has been linked to SDs (Mabuchi et al., 2003). Analysis through the ClinVar database indicates the *COMP* mutation c.1255-5C > T is benign, but whether this variant also contributed to the clinical phenotypes in the family of fetes 13 awaits further investigation.

Mutations in the type I collagen coding genes (*COL1A1* and *COL1A2*) that affect collagen quantity or structure count to approximately 85% of osteogenesis imperfecta (OI) case (Marini et al., 2017; Tournis and Dede, 2018). Mutations that reduce the synthesis of normal type I collagen (quantitative defect) are usually associated with the milder OI, while mutations that alter the protein structure of collagen molecules (structural defect) mostly caused by substitutions of glycine by different amino acids in the helical domain of type 1 collagen are usually linked with more severe phenotypes (Marini et al., 2017). In the variants identified in the present study including *COL1A1* variant c.1192G > T (p. Gly398Cys) and c.1373G > A (p.Gly458Glu), and *COL1A2* variant c.2422G > T (p. Gly808Glu), a glycine in the helical domain of type 1 collagen was substituted by other amino acids. Thus, these variants may damage type 1 collagen functions and therefore cause SDs. *COL2A1* encodes type II collagen that plays a role in the regulation of intramembranous and endochondral ossification. Heterozygous *COL2A1* mutations are usually associates with a spectrum of dwarfism and skeletal malformation diseases (Zhang et al., 2020). In the present study, 2 novel likely pathogenic mutations c.1358G > T (p.Gly453Val) and c.3571G > A (p.Gly1191Arg), and 1 known pathogenic mutation c.1419 + 5G > T were identified in *COL2A1* from fetus 17 to 19 (Table 1). Collectively, these findings support high genetic variability and also highlight the importance of molecular diagnosis for fetal SDs.

In the present study, 5 fetuses showed an autosomal recessive inheritance pattern. Fetus 21 carried a homozygous mutation of *TRIP11* (c.5056 + 1G > C). Mutations in this gene cause autosomal recessive achondrogenesis type IA (ACG1A, MIM_200600) and autosomal recessive osteochondrodysplasia (MIM_184260). Fetus 23 carried compound heterozygosity for mutations c.1757G > A (p.Arg586His) and c.194delG (p.Gly65Valfs^∗^53) in *LBR.* The former was maternal originated, and the later was *de novo*. Both mutations were firstly reported. Mutations of *LBR* cause autosomal dominant Pelger-Huet anomaly (MIM_169400) and autosomal recessive Greenberg skeletal dysplasia (GRBGD, MIM_215140). The ultrasound testing results of shortening of the limbs, small thorax, skull deformation indicate that case 23 was affected with GRBGD. Case 24 carried two novel heterozygous mutations in *IFT172* including c.4540-5T > A of maternal inheritance and c.1513 C > T (p.Arg505Trp) of paternal inheritance. Mutations in *IFT172* cause autosomal recessive short-rib thoracic dysplasia 10 with or without polydactyly. Case 25 carried two novel heterozygous mutations in *FIG4* including c.2174dupT (p.Leu726Ilefs^∗^7) of maternal inheritance and c.573delG (p. Glu192Lysfs^∗^2) of paternal inheritance. Mutation of *FIG4 causes autosomal recessive Yunis–Varon dysplasia* (MIM_ 216340). Case 26 carried two novel mutations in the *DYNC2H1* gene including c.3133C > T (p. Gln1045^∗^) of maternal inheritance and c. 6809G > A (p. Arg2270Gln) of paternal inheritance. Mutation of *DYNC2H1* causes autosomal recessive Short-rib thoracic dysplasia 3 with or without polydactyly (MIM_613091) (Table 1).

## Conclusion

This study has proved that prenatal WES has a high diagnostic rate for SDs, which improves the clinical management of pregnancies and better inform family planning efforts. This study has also identified novel pathogenic variants for SDs, which broadens the mutation spectrum for this disorder and contributes to clinical consultation and subsequent pregnancy examination. As previously reported, VUS and incidental findings in prenatal genetic diagnosis by WES remains as counseling challenges for prenatal diagnosis for structural anomalies, including SDs. Moreover, large-scale prospective studies in fetuses with sonographically identified SDs will provide further information on the feasibility and potential impact of WES on prenatal counseling and pregnancy management.

## Data Availability Statement

The datasets presented in this study can be found in online repositories. The name of the repository and accession number can be found below: National Center for Biotechnology Information (NCBI) BioProject, https://www.ncbi.nlm.nih.gov/bioproject/, PRJNA747169.

## Ethics Statement

The studies involving human participants were reviewed and approved by the ethics committee of the Hunan Provincial Maternal and Child Health Care Hospital. Written informed consent to participate in this study was provided by the participants’ legal guardian/next of kin. Written informed consent was obtained from the minor(s)’ legal guardian/next of kin for the publication of any potentially identifiable images or data included in this article.

## Author Contributions

HW, YP, and CT conceived the study. XH analyzed the results of prenatal ultrasound testing. SY, JP, JL, and JH analyzed the results of karyotyping and SNP array analyses. YP, XS, and CT analyzed the results of WES and prepared the manuscript. All authors contributed to the article and approved the submitted version.

## Conflict of Interest

XS employed by the Berry Genomics Corporation (Beijing, China). The remaining authors declare that the research was conducted in the absence of any commercial or financial relationships that could be construed as a potential conflict of interest.

## Publisher’s Note

All claims expressed in this article are solely those of the authors and do not necessarily represent those of their affiliated organizations, or those of the publisher, the editors and the reviewers. Any product that may be evaluated in this article, or claim that may be made by its manufacturer, is not guaranteed or endorsed by the publisher.

## Footnotes

1
http://broadinstitute.github.io/picard/

2
https://software.broadinstitute.org/gatk/

3
http://gnomad.broadinstitute.org/

4
http://browser.1000genomes.org

5
http://www.ncbi.nlm.nih.gov/snp

6
http://www.omim.org

7
http://www.ncbi.nlm.nih.gov/clinvar

8
http://www.hgmd.org

9
https://hpo.jax.org/app/
