# Supplementary figures and images for: Whole Exome Sequencing Analysis in Fetal Skeletal Dysplasia Detected by Ultrasonography: An Analysis of 38 Cases
Source: Front Genet. 2021 Sep 10;12:728544. doi: 10.3389/fgene.2021.728544 (PMC8461062; doi:10.3389/fgene.2021.728544)

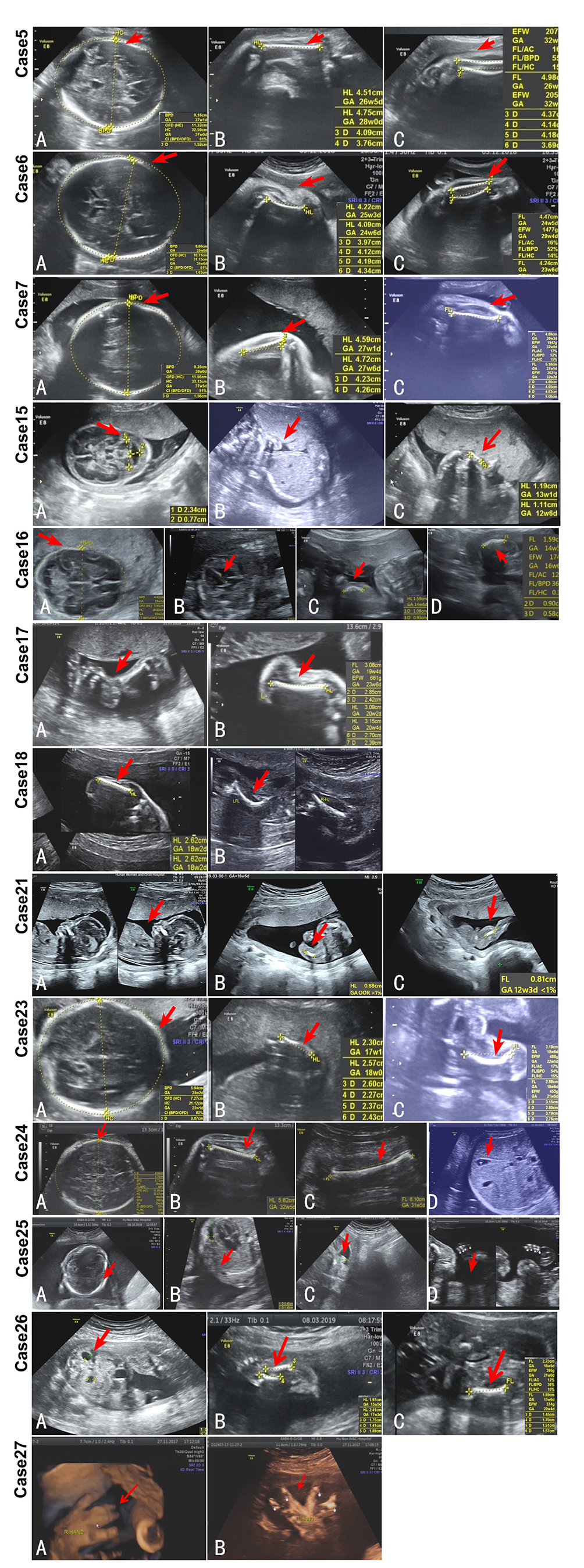

Supplement: Supplementary file 1 [file Image_1.TIF]
