# Supplementary material for: Whole Exome Sequencing Analysis in Fetal Skeletal Dysplasia Detected by Ultrasonography: An Analysis of 38 Cases
Source: Front Genet. 2021 Sep 10;12:728544. doi: 10.3389/fgene.2021.728544 (PMC8461062; doi:10.3389/fgene.2021.728544)
Supplement: Supplementary file 2 [file Data_Sheet_1.docx]

Supplementary Figure 1**.** Ultrasound results of 13 fetuses with novel mutations.

Case 5: skull deformation and out of flatness (A), shortened humerus (B), shortened femur(C).

Case 6:skull deformation and out of flatness(A), shortened humerus (B), shortened femur(C)

Case 7: skull deformation and out of flatness(A), shortened humerus (B), shortened femur(C)

Case15: skull deformation and out of flatness(A), narrow thorax, collapse of sternum (B) shortened humerus(C)

Case16: skull deformation and out of flatness(A), ventricular septal defect(B), shortened humerus (C), shortened femur(D)

Case17: bilateral foot inversion(A), shortened humerus (B)

Case18: shortened humerus (A), curved femur(B)

Case21: narrow thorax, collapse of sternum(A), shortened humerus (B) , shortened femur(C)

Case23: skull deformation and out of flatness, narrow thorax narrow (A), shortened humerus (B), shortened and curved femur(C)

Case24: skull deformation and out of flatness（A, shortened humerus (B) , shortened femur(C), gallbladder echo enhancement(D)

Case25:Scaphocephaly(A), pericardial effusion(B), absence of bilateral thumbs(C), hypospadias(D)

Case26: bilateral renal pelvis and collection system expansion（A）,shortened humerus (B), shortened and curved of femur(C)

Case27: syndactyly of right hand(A), split hand malformation(B)
